# A pilot study on midazolam sedation for murine echocardiography: A potential alternative to isoflurane anesthesia and awake imaging

**DOI:** 10.14814/phy2.70617

**Published:** 2025-11-13

**Authors:** Bart Jacobs, Sarah Derde, Lies Langouche, Jan D'hooge, Nadia Salerno, Sandro Queirós, Greet Van den Berghe, Annette Caenen, Jan Gunst

**Affiliations:** ^1^ Clinical Division and Laboratory of Intensive Care Medicine, Department of Cellular and Molecular Medicine KU Leuven Leuven Belgium; ^2^ Department of Cardiovascular Sciences, Leuven KU Leuven Leuven Belgium; ^3^ Department of Experimental and Clinical Medicine Magna Graecia University Catanzaro Italy; ^4^ Life and Health Sciences Research Institute, School of Medicine University of Minho Braga Portugal; ^5^ 2Ai‐School of Technology IPCA Barcelos Portugal

**Keywords:** awake imaging, cardiac function, echocardiography, isoflurane anesthesia, mice, midazolam sedation

## Abstract

Isoflurane anesthesia is often used to facilitate murine echocardiography, but can suppress cardiac function. Awake imaging avoids pharmacological interference, but can induce sympathetic activation. In this Midazolam sedation. Parameters were compared using repeated‐measures ANOVA. Midazolam enabled imaging without overt stress behavior. Compared to midazolam, heart rate was similar under isoflurane and higher while awake (*p* ≤ 0.01). End‐systolic volume was larger under isoflurane and smaller while awake; stroke volumes remained similar across conditions. Global longitudinal and circumferential strain were less negative under isoflurane (p = 0.03) but similar during awake imaging, while radial strain was higher during awake imaging. Peak longitudinal strain rate was less negative under isoflurane (*p* ≤ 0.01) and more negative while awake (*p* = 0.05). Early diastolic strain rate was similar under isoflurane and lower while awake (*p* = 0.02). In conclusion, cardiac function was most depressed under isoflurane and most enhanced during awake imaging, likely stress‐driven. Murine echocardiography under midazolam sedation was feasible, yielding better function than isoflurane anesthesia, closer to awake imaging but without overt handling stress. These findings require further validation across disease models, sexes, and strains.

## Graphical abstract

This pilot study tested midazolam sedation (C) for murine echocardiography. It yielded better cardiac function than isoflurane anesthesia (B), with values closer to awake imaging (A) but without handling stress.

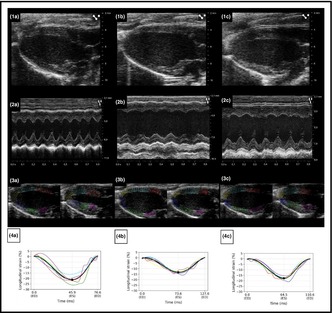

## 1 INTRODUCTION

Echocardiography is a widely used, non‐invasive tool to assess cardiac function in mouse models of cardiovascular disease (Riehle & Bauersachs, 2019). Current guidelines published recommend the use of anesthesia as the primary approach for echocardiographic imaging in mice (Lindsey et al., 2018). Anesthesia facilitates standardization and reduces variability caused by movement and sympathetic activation. Yet, most anesthetics have dose‐dependent effects on cardiac function and/or vessel tone, which could theoretically confound echocardiographic findings. An alternative technique is the use of awake imaging. However, this approach may induce sympathetic stress and requires highly skilled technical staff members to achieve imaging within a reasonable timeframe, which may limit its practical implementation (Lindsey et al., 2018).

The potential usefulness of sedation as an intermediate strategy between full anesthesia and awake imaging has not been thoroughly investigated in mice and is not explicitly addressed in the existing AJP guidelines (Lindsey et al., 2018). Midazolam—a short‐acting benzodiazepine that can be administered subcutaneously or intraperitoneally—has been used safely in behavioral and cognitive research in mice, producing short‐term anxiolysis, sedative‐motor effects, and reduced avoidance behavior, while not causing lasting cognitive deficits (Jovita‐Farias et al., 2023; Valentim et al., 2013). In murine echocardiography, midazolam has most often been used in combination with other anesthetics such as ketamine (Roth et al., 2002; Schaefer et al., 2005) and less frequently as monotherapy (Berry et al., 2009; Tan et al., 2003; Weiss et al., 2006). Yet, its application as a stand‐alone sedation strategy has not been systematically described.

The primary aim of this technical report was to evaluate the feasibility of conducting echocardiography under subcutaneous midazolam monotherapy and to compare the physiological state of mice with this approach to standard isoflurane anesthesia and awake scanning. The goal was to assess whether midazolam sedation could facilitate a calm, minimally stressed physiological state without the need for prior habituation, potentially providing an alternative scanning strategy to isoflurane and awake scanning. To this purpose, we systematically evaluated echocardiographic function parameters—including heart rate, ventricular volumes, and ejection fraction, myocardial strain, and strain rate—across the three imaging conditions in healthy male C57BL/6J mice.

## 2 MATERIALS AND METHODS

### 2.1 Study design and animals

Six male C57BL/6J mice, aged 24 weeks, were obtained from Janvier Labs (Le Genest‐Saint‐Ilse, France). Mice weighed 30.18 ± 1.81 g (data obtained in 5/6 animals). The protocol was approved by the Animal Ethics Committee of KU Leuven (CMM‐163/2022). The study adhered to the European Union Directive 2010/63/EU on the protection of laboratory animals and complied with the ARRIVE guidelines (Kilkenny et al., 2010). Mice were housed in type II filter‐top cages, with two animals per cage in a controlled environment (12 h light/12 h dark cycle, 27°C, relative humidity maintained according to facility guidelines). Standard chow (V1535‐000, ssniff, Soest, Germany) and tap water were provided ad libitum. Each mouse underwent three separate echocardiography sessions on three consecutive days, each separated by a 24‐h washout period to minimize potential carryover effects: (Riehle & Bauersachs, 2019) awake, (Lindsey et al., 2018) under isoflurane anesthesia, and (Jovita‐Farias et al., 2023) under midazolam sedation. The scanning sequence was randomized for each pair of mice, using three different sequences. The chests of the mice were shaved 3 days prior to the start of the echocardiography sessions to ensure optimal image quality.

### 2.2 Echocardiography procedures

All echocardiographic acquisitions were performed by the same two researchers (BJ and SD) throughout the study: one investigator consistently handled the probe and held the animal (BJ), while the other consistently operated the ultrasound system (SD).

#### 2.2.1 Awake imaging

Mice were gently restrained by the scruff of the neck and light tail clamping. Echocardiography was performed while the animal remained awake, using pre‐warmed ultrasound gel. Awake imaging was performed without prior habituation or training.

#### 2.2.2 Echocardiography under isoflurane anesthesia

Mice were placed on a heating pad in an induction box containing 3% isoflurane in 100% oxygen (Iso‐Vet; Piramal Critical Care BV, Voorschoten, The Netherlands). After induction, the mice were transferred to a heated platform where their limbs were gently secured to electrodes using electrode gel and adhesive tape, and their snouts were positioned within a cone for isoflurane administration. Isoflurane was then reduced to 1% and adjusted as necessary during image acquisition. Heart rate was monitored, aiming for >450 bpm, and mice were kept normothermic between 36°C and 37.5°C using the heating pad and a heating lamp. Post‐procedure, mice were placed in a warmed recovery cage until fully awake.

#### 2.2.3 Echocardiography under midazolam sedation

Each mouse received a subcutaneous injection of 0.15 mg of midazolam (1 mg/mL; Mylan BVBA, Hoeilaart, Belgium), equivalent to approximately 5 mg/kg. Injections were administered in the dorsal neck region without additional restraint, keeping handling stress to a minimum. After 5 min in a warmed cage to allow the drug's maximal effect, mice were gently restrained by the scruff of the neck and lightly held at the tail under a heating lamp. Temperature was measured post‐procedure to confirm normothermia and ensure no hypothermia occurred during the session. Post‐procedure, animals were returned to a warmed recovery cage.

ECG monitoring was only performed during isoflurane anesthesia. In awake and midazolam conditions, manually holding the animal precluded the use of limb electrodes and prevented positioning the animals on the heating platform with integrated electrode contacts.

Similarly, continuous core temperature monitoring using a rectal probe was only applied during isoflurane anesthesia. In midazolam‐sedated mice, occasional spontaneous movement could pose a potential risk of probe displacement or injury. Therefore, only a single‐point rectal temperature measurement was performed post‐procedure, when both researchers were available to assist. In awake animals, no temperature measurement was performed, as they were at lower risk of procedure‐related hypothermia, and probe placement could have risks.

### 2.3 Echocardiographic measurements

B‐mode loops were obtained in the parasternal long‐ and short‐axis views, while M‐mode images were acquired in the parasternal short‐axis view using the Vevo 2100 imaging system with the MS400 probe (FUJIFILM VisualSonics, Toronto, Canada). All acquired images were stored, and data extraction was performed blinded for randomization. All measurements and calculations were performed in VevoLab (FUJIFILM VisualSonics, Toronto, Canada). End‐diastolic volume (EDV), end‐systolic volume (ESV), ejection fraction (EF), stroke volume (SV), heart rate (HR), and cardiac output (CO) were measured or derived using the LV trace tool in parasternal long‐axis views and M‐mode images in short‐axis view. HR during the awake condition and under midazolam sedation was calculated by averaging the time intervals between end‐diastolic and end‐systolic markers across at least three consecutive cardiac cycles in M‐mode short‐axis views. Speckle tracking was used to calculate global longitudinal strain (GLS), global circumferential strain (GCS), and global radial strain (GRS). GLS was derived from parasternal long‐axis views, while GCS and GRS were derived from parasternal short‐axis views, all using mid‐myocardial speckle tracking. Strain rates, including peak longitudinal strain rate (PLSR) and early diastolic strain rate (EDSR), were also calculated based on mid‐myocardial speckle tracking from parasternal long‐axis views. Conventional measurements were performed in Vevo LAB®, while strain and strain‐rate metrics were derived from speckle tracking using the Medical Image Tracking Toolbox (MITT) (Queiros et al., 2018).

### 2.4 Statistical analysis

All data are presented as mean ± standard deviation (SD). A repeated measures ANOVA was used to compare the three conditions (awake, midazolam sedation, isoflurane anesthesia), with Tukey's HSD used for post‐hoc pairwise comparisons where appropriate. We performed multivariable regression analyses to assess the potential impact of the randomized investigation sequence on the measured echocardiographic parameters. Models were adjusted for the echocardiography condition (awake, isoflurane, or midazolam) and for the investigated mouse to capture interindividual variability. A *p* ≤ 0.05 was considered significant.

## 3 RESULTS

### 3.1 Duration of the procedures and behavioral observations

The mean duration of echocardiography was 12 ± 6 min under isoflurane anesthesia, 9 ± 3 min for awake imaging, and 13 ± 2 min under midazolam sedation (including the 5‐min waiting period after midazolam injection).

Mice under isoflurane anesthesia were easy to handle once sedation was induced. However, adjustments to the isoflurane dose were occasionally required to maintain optimal sedation levels during imaging, for example, in case of bradycardia or bradypnea. Post‐procedure recovery occurred rapidly, typically within minutes. Awake mice exhibited agitation during echocardiography, including escape behaviors, which prolonged the procedure. Echocardiography under midazolam sedation was feasible. The mice remained calm when held in the hand, showing no inclination to bite or escape. Post‐procedure, the mice appeared mildly sedated but spontaneously mobile, and all sedative effects appeared fully resolved within 4 h.

In isoflurane‐anesthetized mice, occasional adjustments of the heating pad and/or lamp were required to maintain core temperature within the target range (36.0°C–37.5°C). In awake animals, no temperature measurements were performed due to potential risks associated with rectal probe placement in non‐sedated mice. In the midazolam group, four animals were confirmed normothermic post‐procedure (36.0°C–37.5°C). In one animal, rectal probe insertion was unsuccessful at the first attempt and not repeated to minimize potential discomfort. In another, mild hypothermia (34.1°C) was recorded, which was attributed to insufficient heating lamp coverage. Notably, this mouse did not show outlying functional values compared to the group mean.

### 3.2 Echocardiographic assessment

**FIGURE 1:**
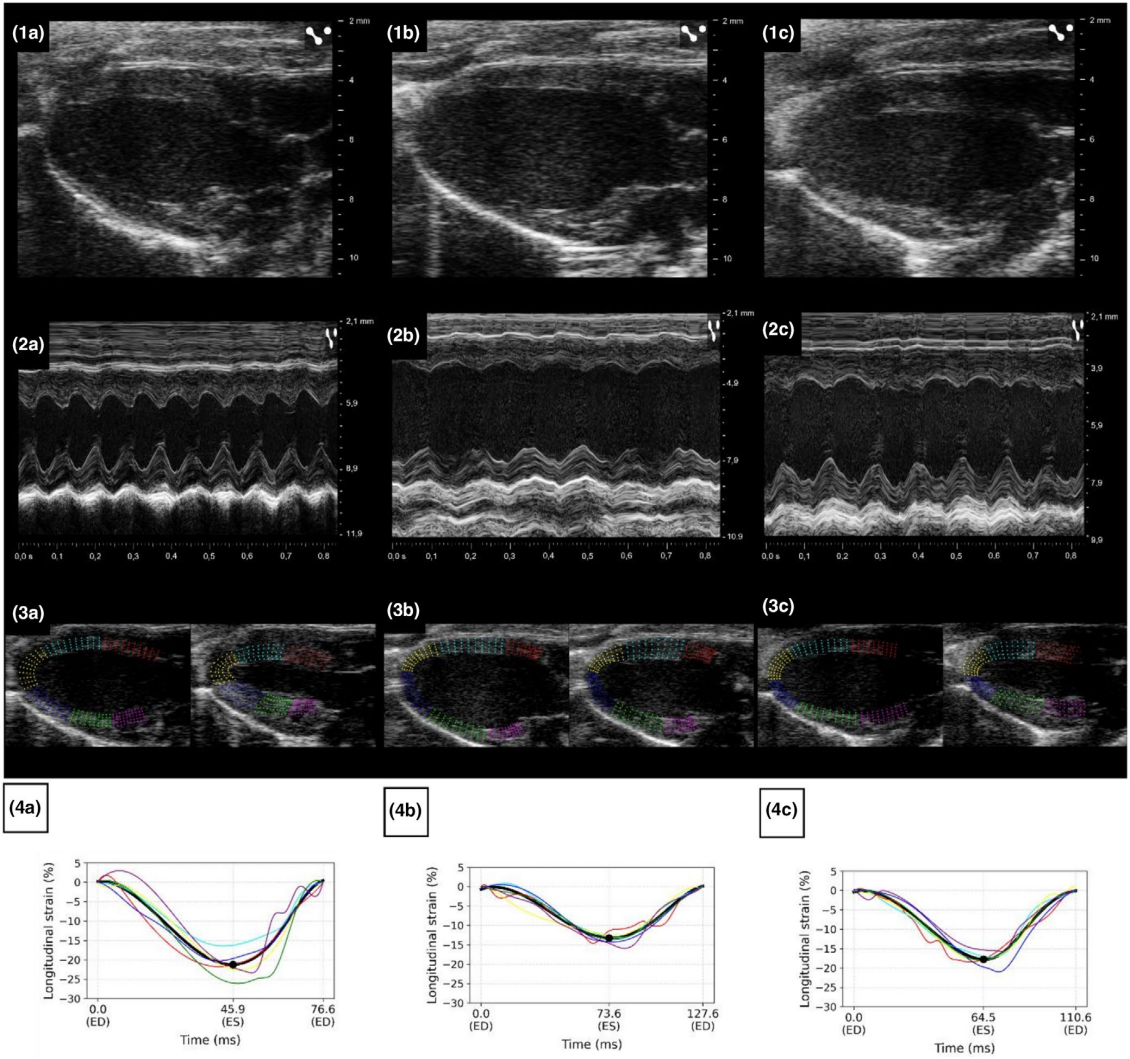
Representative parasternal long‐axis, M‐mode, myocardial wall tracking, and global longitudinal strain images under awake, isoflurane, and midazolam conditions in the same mouse. Parasternal long‐axis B‐mode images at end‐diastole (row 1), M‐mode recordings at the mid‐ventricular level (row 2), illustrative myocardial wall tracking at end‐diastole (ED) and end‐systole (ES) based on endocardial and epicardial border tracings (row 3), and corresponding global longitudinal strain curves (row 4) are shown for the same mouse during awake imaging (a), isoflurane anesthesia (b), and midazolam sedation (c). End‐diastolic volume appears largest under isoflurane and smallest during awake imaging; midazolam shows no clear visual difference from the awake condition. Systolic excursion appears lowest under isoflurane, intermediate under midazolam, and highest in the awake state. Global longitudinal strain curves (black) show the least deformation under isoflurane, and more negative peak values under midazolam and the awake condition.

Figure 1 shows representative parasternal long axis images, M‐mode recordings, and illustrative myocardial wall tracking with corresponding strain curves for the same mouse under awake, isoflurane, and midazolam conditions. End‐diastolic volume appeared largest under isoflurane and smallest during awake imaging, while midazolam showed no clear visual difference from the awake condition. M‐mode recordings showed reduced systolic excursion under isoflurane compared to both awake and midazolam conditions, with midazolam showing an intermediate pattern. Global longitudinal strain curves demonstrated the least negative peak values under isoflurane, with more negative values observed during midazolam sedation and awake imaging.

**FIGURE 2:**
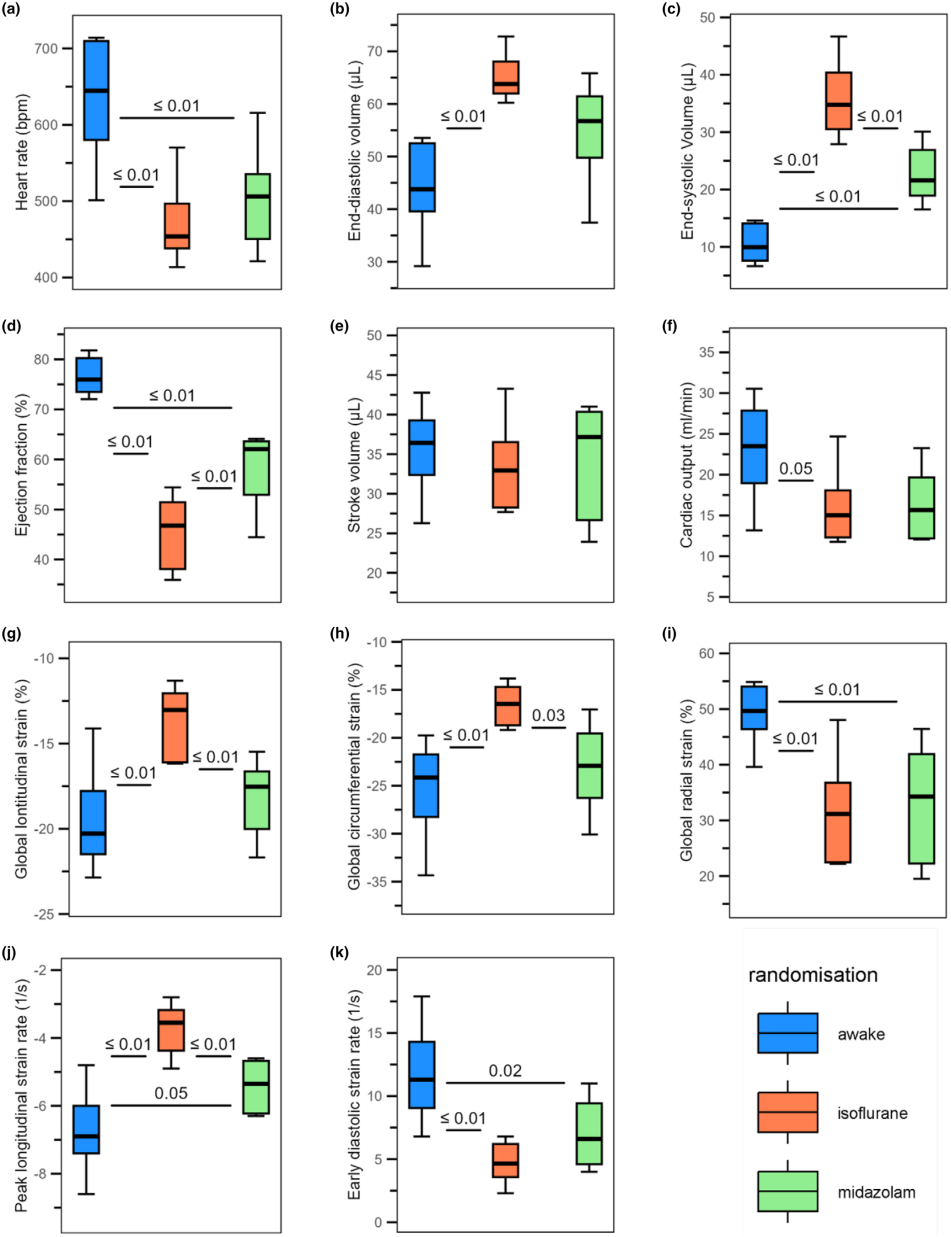
Boxplot representation of echocardiographic measurements across scanning conditions. Boxplots illustrating the effects of awake imaging (blue), isoflurane anesthesia (orange), and midazolam sedation (green) in six mice on (a) heart rate, (b) end‐diastolic volume, (c) end‐systolic volume, (d) ejection fraction, (e) stroke volume, (f) cardiac output, and strain‐based parameters: (g) global longitudinal strain, (h) global circumferential strain, (i) global radial strain, (j) peak longitudinal strain rate, and (k) early diastolic strain rate. The box represents the interquartile range (Q1–Q3) with the median, while the whiskers extend to the minimum and maximum values. Statistical comparisons were performed using repeated measures ANOVA, followed by Tukey's HSD for post‐hoc pairwise comparisons.

Table 1, Figure 2 show the echocardiographic parameters in the three scanning conditions. Compared to midazolam, HR (503 ± 65 bpm) was similar under isoflurane (*p* = 0.64) and higher in the awake condition (+27%, *p* ≤ 0.01). EDV (55.1 ± 9.7 μL) tended to be higher under isoflurane (+18%, *p* = 0.11) and tended to be lower in the awake condition (−20%, *p* = 0.07), while ESV (22.6 ± 4.8 μL) was significantly higher under isoflurane (+58%, *p* ≤ 0.001) and significantly lower in the awake condition (−53%, *p* ≤ 0.01). EF under midazolam (58.7 ± 7.6%) was lower under isoflurane (mean difference = −13%, p ≤ 0.01) and higher in the awake condition (mean difference = +18%, p ≤ 0.001). CO under midazolam (16.2 ± 4.3 mL/min) was similar under isoflurane (*p* = 0.98) and tended to be higher in the awake condition (+42%, *p* = 0.07), while SV (34.5 ± 7.1 μL) was similar under the three scanning conditions. Compared to midazolam, GLS and GCS (−18.1 ± 2.2% and −23.1 ± 4.4%) were less negative under isoflurane (mean difference = +4.5%, *p* ≤ 0.01; mean difference = +6.5%, *p* = 0.03) and were similar in the awake condition (*p* = 0.5 and *p* = 0.64, respectively). Compared to midazolam, GRS (33.0 ± 10.3%) was similar under isoflurane (*p* = 0.94) and was higher in the awake condition (+16.4%, *p* ≤ 0.01). PLSR (−5.4 ± 0.7 s^−1^) was less negative under isoflurane (+31%, p ≤ 0.01) and tended to be more negative in the awake condition (−25%, *p* = 0.05). EDSR (7.0 ± 2.6 s^−1^) was similar to isoflurane (*p* = 0.36) and was higher in the awake condition (+68%, *p* = 0.02).

**TABLE 1:** Effect of scanning conditions on echocardiographic measurements.

Echocardiographic measurement	Awake	Isoflurane	Midazolam	Midazolam vs. isoflurane	Isoflurane vs. awake	Midazolam vs. awake
Heart rate (beats/min)	637 ± 79	468 ± 54	503 ± 65	0.64	≤0.01	≤0.01
End‐diastolic volume (μL)	44.3 ± 8.7	64.9 ± 4.4	55.1 ± 9.7	0.11	≤0.01	0.07
End‐systolic Volume (μL)	10.5 ± 3.3	35.6 ± 6.5	22.6 ± 4.8	≤ 0.01	≤0.01	≤ 0.01
Ejection fraction (%)	76.6 ± 3.7	45.5 ± 7.0	58.7 ± 7.6	≤0.01	≤0.01	≤0.01
Stroke volume (μL)	35.7 ± 5.5	33.3 ± 5.6	34.5 ± 7.1	n/a	n/a	n/a
Cardiac output (ml/min)	23.1 ± 6.0	15.8 ± 4.6	16.2 ± 4.3	0.98	0.05	0.07
Global longitudinal strain (%)	−19.6 ± 3.0	−13.7 ± 2.0	−18.1 ± 2.2	≤0.01	≤0.01	0.5
Global circumferential strain (%)	−25.2 ± 5.0	−16.6 ± 2.0	−23.1 ± 4.4	0.03	≤0.01	0.64
Global radial strain (%)	49.4 ± 5.4	31.4 ± 9.4	33.0 ± 10.3	0.94	≤0.01	≤0.01
Peak longitudinal strain rate (1/s)	−6.8 ± 1.2	−3.7 ± 0.7	−5.4 ± 0.7	≤0.01	≤0.01	0.05
Early diastolic strain rate (1/s)	11.7 ± 3.7	4.7 ± 1.6	7.0 ± 2.6	0.36	≤0.01	0.02

*Note*: The table presents mean ± standard deviation values of echocardiographic measurements from six mice under three conditions: awake scanning, isoflurane anesthesia, and midazolam sedation (first three columns). The last three columns show *p*‐values from Tukey post‐hoc tests for pairwise comparisons, conducted only when repeated measures ANOVA indicated a significant effect (*p* ≤ 0.05); otherwise, post‐hoc tests were not performed (“n/a”).

Regression analysis identified scanning condition (midazolam sedation, awake imaging, or isoflurane anesthesia) as a significant factor (*p* < 0.05) for all parameters that differed between groups. The randomized imaging sequence showed no independent effect, and only individual mouse variability was associated with PLSR.

## 4 DISCUSSION

In this technical report, we described a procedure to perform echocardiography in healthy, 24‐week‐old male C57BL/6J mice under subcutaneous midazolam sedation and found that such procedure was feasible. Functional parameters under midazolam sedation were generally intermediate between those observed under isoflurane anesthesia and awake imaging. This may be explained by minimizing both sympathetic activation and pharmacological cardiosuppression. Midazolam may therefore offer a practical alternative to established imaging approaches. Nevertheless, further validation is needed to confirm generalizability.

Echocardiography under midazolam sedation was technically feasible in all animals and did not require advanced setup or invasive instrumentation. The mice remained calm during imaging, and none exhibited escape behavior. Subcutaneous administration in the dorsal neck region required only minimal handling and restraint. One animal developed mild hypothermia, attributed to suboptimal heating lamp positioning. Although this might have confounded the echocardiographic assessment, the measured parameters were similar to those in other animals scanned under midazolam sedation, making bias unlikely. This underscores that careful lamp positioning is essential, as closer placement increases the risk of burns. Midazolam lacks the flexibility of inhaled agents such as isoflurane, where anesthetic depth can be titrated. Nevertheless, the availability of flumazenil as a specific antagonist offers a potential means of reversal to be considered in cases of prolonged sedation or hypothermia.

The goal of murine echocardiography is to assess cardiac function in a relaxed and not overly sympathetically driven state. In general, cardiac function parameters under midazolam sedation were between those observed in awake scanning and those observed with isoflurane anesthesia. Compared to awake scanning, midazolam sedation was associated with lower heart rates, higher ESV, and decreased systolic function parameters (EF, GRS, EDSR), alongside a more negative peak longitudinal strain rate (PLSR). Midazolam may have prevented stress‐induced sympathetic activation observed in awake imaging, although we cannot exclude a suppressive effect of midazolam on cardiac function (Tan et al., 2003). Compared to isoflurane, midazolam sedation was associated with better cardiac function, including higher EF and more negative myocardial strain and strain rate values (GLS, GCS, and PLSR), in the context of a similar heart rate. This may reflect less pharmacologic depression or a more balanced autonomic state, though these hypotheses require further validation. Future studies should assess whether midazolam sedation is non‐inferior or superior to isoflurane.

Compared to our data, previous studies in C57BL/6J mice under isoflurane anesthesia generally demonstrated lower volumes (EDV and ESV) and parameters consistent with better cardiac function, including higher EF and more negative GLS, GCS, and PLSR (Lindsey et al., 2018; Wang et al., 2015; Wang et al., 2023; Zacchigna et al., 2021). Similarly, under midazolam sedation, one prior study reported a substantially higher heart rate and EF in C57BL/6 mice compared to our cohort, despite an identical midazolam dose (0.15 mg SC) (Berry et al., 2009). These differences with previous studies may be explained by differences in age or substrain, timing of measurement, or variation in strain analysis software. In the current study, animals were older than in previous studies reporting strain values (24 weeks vs. 8–10 weeks), which could at least partially explain differences seen in our cohort (Wang et al., 2015; Wang et al., 2023).

In this study, we included only healthy animals. The magnitude of the effect of the imaging condition on cardiac functional parameters likely differs in disease conditions or genetically modified models. States of reduced cardiac function could be anticipated to confer a greater susceptibility to an altered sympathetic activation status and pharmacological cardiosuppression, which requires further investigation. In this regard, non‐reperfused myocardial infarction mice models show mortality rates of 10%–40% (Lindsey, Brunt, et al., 2021) and may be particularly sensitive to anesthetic‐induced hemodynamic and respiratory depression, increasing the risk of oversedation‐related mortality. Similarly, obesity may increase the risk of complications related to oversedation (Seyni‐Boureima et al., 2022).

Our study inherently has limitations. In this pilot study, we only included 24‐week‐old male C57BL/6J mice to reduce biological variability within a limited sample size, and measurements were performed by one trained operator to avoid interobserver variability. Future studies should investigate whether our findings are generalizable to animals of different age, sex, and strain, and whether the timing and operator experience affect the results. Although male mice are not impacted by cyclic estrogen levels, studies have suggested that male mice may be equally or more susceptible to hormonal fluctuations, handling‐related stress, and cage effects than female mice (Lindsey, LeBlanc, et al., 2021). Second, under midazolam sedation, heart rate was assessed only at a single time point using M‐mode imaging. Use of limb electrodes was not feasible, as mice were not fully anesthetized. Hence, the assessment may not accurately reflect fluctuations throughout the entire imaging procedure. Future adaptations—such as modified adhesive electrodes—may help overcome current limitations in restrained or sedated animals. Third, although data extraction was performed blinded to the randomized scanning sequence, full blinding to imaging condition was not feasible due to the presence of ECG signals exclusively in the isoflurane group. Fourth, we did not include mice who were trained for awake scanning. Prior habituation or training may reduce sympathetic stress. However, even with training, cardiac function in awake mice would likely remain affected by some stress. Consequently, there is no absolute standard for direct comparison between imaging conditions. Fifth, diastolic function was not fully assessed. Although we did assess EDSR, conventional mitral inflow and tissue Doppler measurements of the mitral annulus requiring apical views were not obtained. Finally, the study was exploratory in nature and not formally powered for hypothesis testing. Statistical comparisons should be interpreted with caution, and no definitive physiological conclusions can be drawn.

In conclusion, subcutaneous midazolam sedation allowed echocardiography in 24‐week‐old male C57/BL6J mice without invasive instrumentation, overt stress behaviors, or extensive physical restraint. Cardiac function was overall most depressed under isoflurane anesthesia and most enhanced during awake imaging, the latter likely reflecting stress‐induced sympathetic activation. Midazolam sedation provided a relaxed state, yielded better cardiac function than isoflurane anesthesia, and values closer to awake imaging but without handling stress. It may therefore represent an alternative for murine echocardiography, requiring further validation.

## AUTHOR CONTRIBUTIONS

Bart Jacobs, Sarah Derde, Lies Langouche, Greet Van den Berghe, Jan D'hooge, Nadia Salerno, Annette Caenen, and Jan Gunst participated in the design of the study and drafting the manuscript. Bart Jacobs, Sarah Derde, and Lies Langouche participated in data collection and, together with Jan D'hooge, Nadia Salerno, Annette Caenen, and Jan Gunst, were responsible for the interpretation of the results. Lies Langouche modified and fine‐tuned the Medical Image Tracking Toolbox (MITT) and software to optimize its application for this study. All authors read and approved the final manuscript for publication.

## CONFLICT OF INTEREST STATEMENT

The authors have no conflicts of interest to disclose.

## ETHICS STATEMENT

The content is solely the responsibility of the authors and does not necessarily represent the official views of their affiliated institutions.

## Data Availability

The data that support the findings of this study are available from the corresponding author upon reasonable request.
